# An update of the genera *Idiasta* Foerster and *Rhacalysia* Cameron (Hymenoptera, Braconidae, Alysiinae) and the descriptions of new species from the Neotropical Region

**DOI:** 10.3897/zookeys.976.56751

**Published:** 2020-10-20

**Authors:** Franciélle Dias de Oliveira, Angélica Maria Penteado-Dias

**Affiliations:** 1 Programa de Pós-Graduação em Ecologia e Recursos Naturais, Universidade Federal de São Carlos – UFSCar, CP 676, CEP 13 565–905, São Carlos, SP, Brazil Universidade Federal de São Carlos São Carlos Brazil; 2 Universidade Federal de São Carlos – UFSCar, Departamento de Ecologia e Biologia Evolutiva, CP 676, CEP 13 565–905, São Carlos, SP, Brazil Universidade Federal de São Carlos São Carlos Brazil

**Keywords:** Alysiini, Brazil, parasitic wasp, parasitoid, taxonomy

## Abstract

Taxonomic combinations have been made involving the two genera *Idiasta* Foerster and *Rhacalysia* Cameron. Four new species are described from Brazil: *Idiasta rupina***sp. nov.**, *Rhacalysia ampla***sp. nov.**, *Rhacalysia jatai***sp. nov.**, and *Rhacalysia monteiroi***sp. nov.** Dichotomous identification keys to the Neotropical species of *Idiasta* and *Rhacalysia* are provided. *Phaenocarpa delicata* Papp, 1969 is included in *Rhacalysia* and is a new combination.

## Introduction

*Idiasta* Foerster, 1863 and *Rhacalysia* Cameron, 1910 are both genera of the tribe Alysiini (Braconidae, Alysiinae). Members of *Idiasta* possess the largest set of plesiomorphic characteristics within the *Phaenocarpa* complex (Wharton 2002). The taxon was revised by Königsmann (1969), Papp (1969), Fischer (1975), and Wharton (1980, 2002). The biology of *Idiasta* is poorly known since its members are rarely collected (Wharton 2002). There is a single host record in the literature to *Idiasta euryzona* Wharton, 1980 associated to puparia of *Lispe* flies (Diptera, Muscidae) (Wharton 1984). The genus *Idiasta* is cosmopolitan, currently with 51 described species (Peris-Felipo 2016; Yu et al. 2016), of which three are in the Neotropical Region: *Idiasta euryzona* Wharton, 1980, *I. dixi* Dix, 2010, and *I. maritima* (Haliday, 1838). In the Neotropical Region *Idiasta euryzona* and *I. maritima* are known from Mexico (Wharton 1980), and *I. dixi* from Colombia (Dix 2010). One new species is described and illustrated from Brazil: *Idiasta rupina* sp. nov.

The genus *Rhacalysia* includes four described species: *Rhacalysia congoensis* Fischer, 1993, *R. delicata* (Papp, 1969) comb. nov., *R. profundinigra* Fischer, 1999, and *R. rufobalteata* Cameron, 1910. The biology of *Rhacalysia* is unknown. *Rhacalysia congoensis* is known from Republic of the Congo (Fischer 1993) and *Rhacalysia profundinigra* and *R. rufobalteata* from India (Fischer 1999). Up to now, *Rhacalysia delicata* is the only species of the genus known from the New World and widely distributed in the Neotropical Region. It is reported from Argentina (Papp 1969), Brazil (Arouca and Penteado-Dias 2009), Colombia (Wharton 1980; Dix 2010), Panama, Peru, and Venezuela (Wharton 1980).

The genus *Rhacalysia* was treated as a synonym of *Idiasta* by Shenefelt (1974), Fischer (1967), Bhat (1979), and Marsh (1979), Wharton (1980) considered it at least to be a subgenus, and finally Fischer (1994, 1999), Ray (1999) and Wharton (2002) treated it as a valid genus.

*Rhacalysia delicata* was originally included in *Phaenocarpa* (Papp, 1969), later attributed to *Idiasta* (Fischer 1975; Wharton 1980), and then transferred to *Rhacalysia* by Fischer (1994) after he examined the male holotype. Peculiarly, *R. delicata* was not included in the key of *Rhacalysia* species by Fischer in 1999. Wharton (2017) pointed to the uncertain generic position of *R. delicata*, but included it in *Idiasta*, as was done by other authors (Braet and van Achterberg 2003; Arouca and Penteado-Dias 2009; Dix 2010). After discovering several additional species reported in this paper, it is obvious that the transfer by Fischer (1994) was correct and we accept the inclusion in *Rhacalysia*. Three new species are described, keyed, and illustrated from Brazil: *Rhacalysia ampla* sp. nov., *R. jatai* sp. nov., and *R. monteiroi* sp. nov.

## Materials and methods

Two specimens of *Idiasta* and eight of *Rhacalysia* were studied. *Idiasta rupina* sp. nov. was collected in a grassland environment characterized as rupestrian grassland (campo rupestre) (Fernandes 2016), in a patch of gallery forest, at the Parque Nacional da Serra da Canastra, São Roque de Minas, Minas Gerais, Brazil, at 1317 m. *Rhacalysia ampla* sp. nov. and *R. monteiroi* were collected in the Parque Nacional da Serra dos Órgãos, Teresópolis, Rio de Janeiro, as follows: *Rhacalysia ampla* sp. nov. at 1236 to 1649 m; *Rhacalysia monteiroi* at 252 to 1482 m. In an area of Atlantic forest, the vegetation of this park is classified as dense ombrophilous forest, and the physiognomies vary according to altitude: low-montane forests occur up to 600–800 m; montane forests are between 600–1600 m; high-montane forests may occur above 1300 m; and altitude fields occur above 1600 m (Rizzini 1979). *Rhacalysia jatai* sp. nov. was collected in Estação Ecológica de Jataí, Luiz Antônio, São Paulo, in a seasonal forest (Coutinho 1978).

Wharton et al. (2017) was used to identify specimens in subfamily and genus. The morphological terminology is based on van Achterberg (1993); terminology for body sculpture follow Eady (1968). The measurements follow Wharton (1977), with additions and modifications by Kula and Zolnerowich (2005, 2008), except for legs, which are as in Peris-Felipo (2016). All the material is deposited in the **DCBU** collection (Departamento de Ecologia e Biologia Evolutiva da Universidade Federal de São Carlos, SP, Brazil).

Digital Scanning Electronic Microscope (SEM) photographs of uncoated specimens were taken with a FEI Quanta 250 SEM in a low vacuum mode. Color digital photographs were taken with a Leica M165C stereomicroscope, using a Leica DFC295 HD camera, LEICA APPLICATION SUITE software version 3.7. ADOBE PHOTOSHOP CS5 Extended version 12.1. was used for minor corrections of images and for the preparation of plates.

## Taxonomic accounts

### Genus Idiasta

Taxon classificationAnimaliaHymenopteraBraconidae

Foerster, 1863

D43E1D34-4697-5E8C-AB1A-A9968F43D8FE

#### Type species.

Idiasta (Alysia) maritima Haliday, 1838

#### Diagnosis.

Mandibles with three teeth, ventral and diagonal ridges well developed. First flagellar segment equal or shorter than second. Fore wing pterostigma broad, discrete, wedge-shaped; 2-SR vein longer than 3-SR. Hind wing with m-cu present, often well developed, M+CU generally equal to or longer than 1-M.

#### Hosts.

Muscidae (Diptera).

#### Distribution.

Cosmopolitan.

### Idiasta rupina sp. nov.

Taxon classificationAnimaliaHymenopteraBraconidae

192739DB-D55F-5997-9352-77890262FE85

http://zoobank.org/5866B8BE-42BC-4683-84DD-B1F489BC711F

Figures 1–7

#### Type material.

***Holotype*** pinned, female, (DCBU 404791) Brazil, Minas Gerais, São Roque de Minas, Parque Nacional da Serra da Canastra, 20°15'15.29"S, 46°25'14.38"W, alt. 1317 m, 05–07.I.2019, rupestrian grassland, Moericke traps, A. S. Soares col. ***Paratype***, male, (DCBU 404792) same data as holotype.

#### Diagnosis.

*Idiasta rupina* is distinct from other Neotropical species in having the eyes glabrous, notauli incomplete, metanotum with high median flange, m-cu of fore wing interstitial, and fore wing cu-a postfurcal.

#### Description.

**Figures 1–7.**
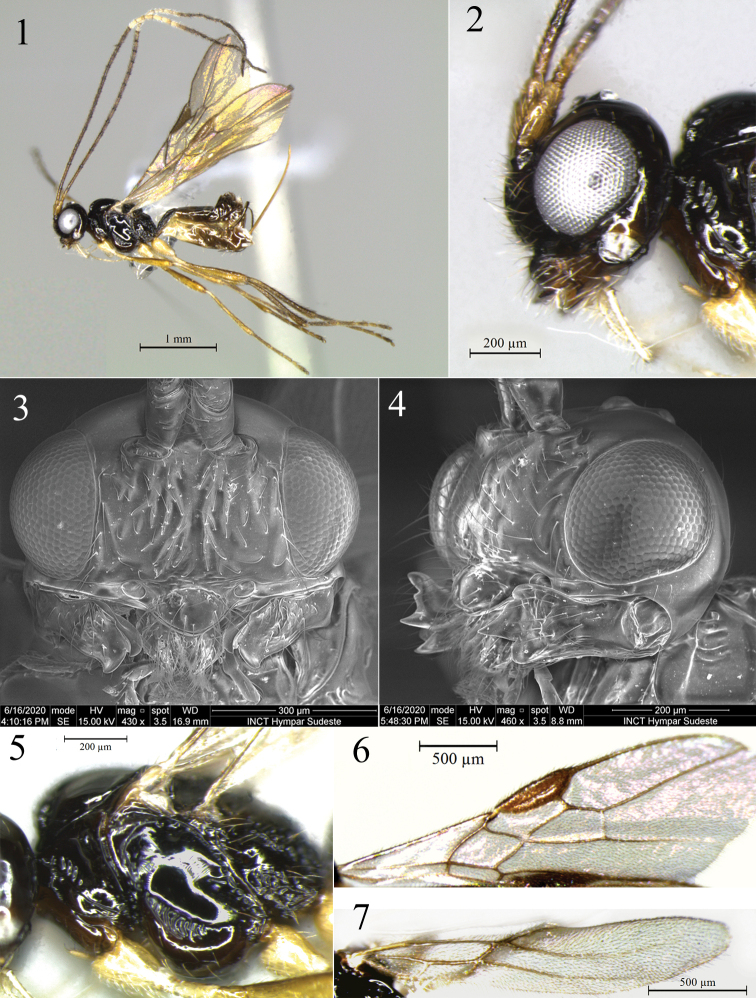
*Idiasta rupina* sp. nov. (**1, 2, 5–7** female, holotype **3** and **4** male, paratype) **1** habitus, lateral view **2** head, lateral view **3** head, anterior view **4** head, lateral view showing mandible **5** mesosoma, lateral view **6** fore wing **7** hind wing.

**Female** (holotype) (Fig. 1). ***Length*.** Body 2.65 mm; fore wing 2.7 mm; hind wing 1.9 mm.

***Head*.** 1.6× as wide as long; 1.8× as wide as face, 1.6× as wide as mesosoma, 3.3× as wide as apex of first metasomal tergite; slightly wider at eyes than temples in dorsal view. Eye glabrous, 1.2× as high as wide, 2.6× as wide as temple in lateral view (Fig. 2). Occiput, vertex, frons and temples smooth, with some sparse setae. Face 1.7 × as wide as high, setose; slightly rugulose above clypeus (Figs 3, 4). Epistomal sulcus well defined but almost shallow, slightly rugulose. Clypeus protruding, smooth, setose, 1.5× as wide as high; lateral margin of clypeus in contact with paraclypeal fovea (Fig. 3). Malar space ca. 1/10 eye height. Paraclypeal fovea occupying 2/7 of distance between lateral margin of clypeus to eye. Mandible 3-dentate (Fig. 4), 1.9× as long as apical width, slightly wider in apex than base; setose, rugulose antero-medially, punctate; diagonal ridge well developed on apical half of mandible, ventral ridge complete; teeth 1 and 2 connected by flange, incision present but unobtrusive; teeth 1 and 3 approximately equal in size, tooth 2 wider and longer than others. Antenna 1.8× as long as body, with 32 flagellar segments. First flagellar segment 3.3× as long as wide; second flagellar segment 8.2× as long as wide, 2.5× length of first segment; third flagellar segment 6.5× as long as wide, 1.8× length of first. Maxillary palp 1.7× as long as head height.

***Mesosoma*.** 1.4× as long as high, 1.9× as long as wide, 1.7× as high as head. Pronotum in dorsal view with small but distinct pronope, crenulate in posterior margin; in lateral view, crenulate in upper middle area. Mesoscutum 1.1× as wide as long, scattered setae present along notauli. Notauli deep, crenulate anteriorly, absent posteriorly. Mesoscutal pit deep, slightly elongate, occupying a little less than 1/5 extent of mesoscutum. Scutellar sulcus 2.5× as wider as long, with well-developed mid ridge and some weak ridges at posterior margin of lateral areas. Scutellar disc smooth, setiferous; parascutellar area weakly rugulose posteriorly, with setae near scutellar sulcus. Metanotum setiferous anteriorly, in dorsal view rugose medially, depressed lateral fields crenulate; mid ridge complete and two well-developed median lateral ridges; metanotum in lateral view with high median flange. Mesopleuron with scattered setae in ventral area, antero-basal margin crenulate towards anterior subalar area; posterior margin crenulate. Precoxal sulcus deep, long, widely crenulate, separated from posterior margin (Fig. 5). Propodeum with anterior half nearly smooth and median carina; posterior half rugose, including inside areola; areola pentagonal, ca. as long as wide. Metapleuron rugose (except medially) and setose.

***Fore wing*.** Approximately as long as body. Pterostigma 3.4× as long as wide, 2.25× as wide as vein r length; r 0.3× as long as 2-SR, arising distad midpoint of pterostigma; submarginal cell 2.6× as long as high; 2-SR 2.5× as long as r-m, 1.4× as long as 3-SR; 3-SR 2.65× as long as r, 1.8× as long as r-m; SR1 5.15× as long as 3-SR; 2-CU1 1.1× as long as m-cu, this interstitial; cu-a postfurcal by distance less than its length; subdiscal cell closed, nearly parallel-sided; CU1a arising slightly above middle of subdiscal cell (Fig. 6).

***Hind wing*.** With three hamuli, 5.7× as long as wide; vein 1-M 1.15× as long as M+CU, 2.0× as long as 1r-m; m-cu interstitial, spectral (Fig. 7).

***Legs*.** Hind femur 6.35× as long as wide. Hind tibia 11.2× as long as its maximum subapical width, 0.9× as long as hind tarsus. First segment of hind tarsus 1.7× as long as second segment.

***Metasoma*.** First metasomal tergite 1.6× as long as apical width; apex 1.5× as wide as base; strongly strigose surface, dorsal carinae convergent and uniting in basal third, continuing as a distinct median carina to apex, dorsope deep. Ovipositor 1.3× as long as hind tibia, 1.45× as long as mesosoma; straight and strongly directed upwards (Fig. 1). Ovipositor sheath setose.

***Color*.** Dark brown to black. Gena and mandibles brown, mandibles lighter in apical third; scape, pedicel, and basal half of first flagellar segment yellow brown; flagellar segments 2–13 brown, 14–19 white, 20–32 dark brown. Propleuron, ventral mesopleuron, and tegulae brown. Second and third metasomal tergites brownish yellow. Legs yellow, gradually darkening towards apex; coxa and trochanter pale yellow; hind tibia and hind tarsus brown. Wings hyaline, venation and pterostigma brown.

**Male.** Similar to female but body length 2.4 mm; hind wing 2.0 mm; head 3.0× as wide as apex of first metasomal tergite; antenna with 36 flagellar segments; first flagellar segment 3.9× as long as wide; second flagellar segment 7.95× as long as wide, 1.8 × length of first segment; third flagellar segment 7.0× as long as wide, 1.4× length of first segment; maxillary palp 2.4× as long as head height. Mesoscutum slightly longer than wide; scutellar sulcus 2.25× as wide as long. Fore wing with 2-SR vein 1.9× as long as r-m, 1.6× as long as 3-SR; 3-SR 1.2× as long as r-m. Hind wing 1-M 0.8× as long as M+CU, 2.6× as long as 1r-m. Hind femur 5.15× as long as wide; first segment of hind tarsus 1.5× as long as second segment. Antenna brown except yellowish basal half of first flagellar segment; wing venation and pterostigma light brown.

#### Etymology.

The species name refers to the ecosystem from which the studied material was collected.

#### Distribution.

Brazil, State of Minas Gerais, São Roque de Minas, rupestrian grassland.

#### Comments.

*Idiasta rupina* and *I. dixi* are related Neotropical species and share the notauli absent posteriorly, fore wing cu-a postfurcal, and hind wing m-cu not tubular. The color pattern of the body is also similar. However, *Idiasta rupina* can be differentiated by the glabrous eye (with sparse setae in *I. dixi*), high median flange of the metanotum (absent in *I. dixi*), fore wing m-cu interstitial (slightly antefurcal in *I. dixi*). Additionally, *Idiasta rupina* differ in the following quantitative ratios: eye 2.5 × as wide as temple (3.1× in *I. dixi*); maxillary palp ca. twice as long as head height (1.4 × in *I. dixi*); sulcus scutellar 2.5× as wide as long (1.4× in *I. dixi*); fore wing vein 3-SR 2.6 × as long as r (3.5× in *I. dixi*); SR1 5.1× as long as 3-SR (4.5× in *I. dixi*); ovipositor 1.4× as long as mesosoma (ca. 1.0× in *I. dixi*).

### Genus Rhacalysia

Taxon classificationAnimaliaHymenopteraBraconidae

Cameron, 1910

EE8D7934-0C58-51EB-A58A-B3C063501539

#### Type species.

*Rhacalysia rufobalteata* Cameron, 1910

#### Diagnosis.

Enlarged paraclypeal fovea extending to eye. First flagellar segment shorter than second. Fore wing venation complete; 2-RS vein longer than 3-SR, m-cu antefurcal to interstitial. Metasomal terga unsculptured beyond the first tergite, not forming a carapace.

#### Hosts.

Unknown.

#### Distribution.

Afrotropical, Asian, and Neotropical Regions.

### Rhacalysia ampla sp. nov.

Taxon classificationAnimaliaHymenopteraBraconidae

9E40FBEB-6078-599F-B97D-0847919A7C9C

http://zoobank.org/543C6C87-1523-4941-8513-26A6C4D42200

Figures 8–11 , 12–18

#### Type material.

***Holotype*** pinned, female (DCBU 361839) Brazil, Rio de Janeiro, Teresópolis, Parque Nacional da Serra dos Órgãos, 22°26'57"S, 43°00'13"W, alt. 1236 m, XI.2015, dense ombrophilous forest, Malaise trap, R. F. Monteiro col. ***Paratypes***, females (3), (DCBU 358123) 22°26'57"S, 43°00'13"W, alt. 1236 m, III.2015, Malaise trap, R. F. Monteiro col.; (DCBU 360613) 22°27'03"S, 43°00'54"W, alt. 1649 m, IV.2015, Malaise trap, R. F. Monteiro col.; (DCBU 404793) 22°27'03"S, 43°00'54"W, alt. 1649 m, I.2015, Malaise trap, R. F. Monteiro col.

#### Diagnosis.

*Rhacalysia ampla* can be differentiated from other species of genus by the notauli incomplete, precoxal sulcus sculptured only in anterior fourth, fore wing with m-cu interstitial, CU1a arising below middle of subdiscal cell, and hind wing with three hamuli.

#### Description.

**Figures 8–11.**
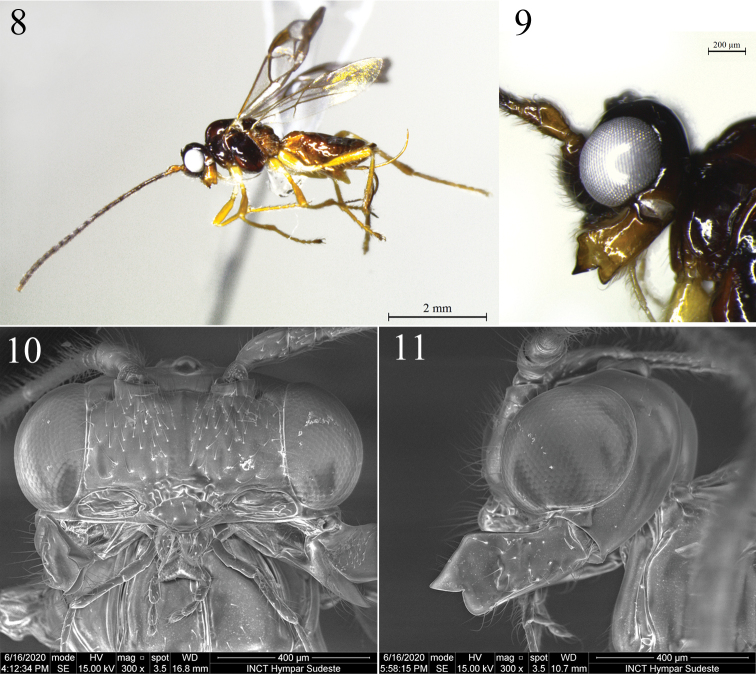
*Rhacalysia ampla* sp. nov. (females, paratypes) **8** habitus, lateral view **9** head, lateral view **10** head, anterior view **11** head, lateral view showing mandible.

**Female** (Fig. 8). ***Length*.** Body 3.1–4.0 mm; fore wing 3.4–4.4 mm; hind wing 2.8–3.45 mm.

***Head*.** 1.5–1.85× as wide as long; 1.7× as wide as face, 1.5–1.6× as wide as mesosoma, ca. 2.2× as wide as apex of first metasomal tergite; slightly wider at eyes than temples in dorsal view. Eye glabrous, 1.0–1.1× as high as wide, 2.9–3.0× as wide as temples in lateral view (Fig. 9). Occiput, vertex, and temples smooth, with some sparse setae. Frons occasionally with weak pit mesally. Face 2.1–2.2× as wide as high, setose; low mid ridge dorsally, rugulose above clypeus (Fig. 10). Epistomal sulcus deep, crenulate. Clypeus protruding, smooth to rugulose, setose (setae as long as wide clypeus), 1.6–2.0× as wide as high; lateral margin of clypeus does not contact with paraclypeal fovea. Malar space short, 1/13 eye height. Paraclypeal fovea enlarged to form broad groove extending to eye (Fig. 10).

Mandible 3-dentate (Figs 9, 11), 1.7–1.9× as long as apical width, apex 1.2–1.3× as wide as base; setose, slightly rugose antero-medially; diagonal ridge well developed on apical half of mandible, relatively displaced to ventral margin, and ventral carina present in basal half; teeth 1 and 2 connected by flange, indistinct incision; tooth 3 wider than tooth 1; tooth 2 wider and longer than others. Antenna 1.7× as long as body, with 38 flagellar segments (holotype). First flagellar segment 3.5–3.8× as long as wide; second flagellar segment 6.3–6.9× as long as wide, 1.7–2.0× length of first segment; third flagellar segment 5.4–5.9× as long as wide, 1.5–1.8× length of first segment. Maxillary palp 1.9–2.0× as long as head height.

**Figures 12–18.**
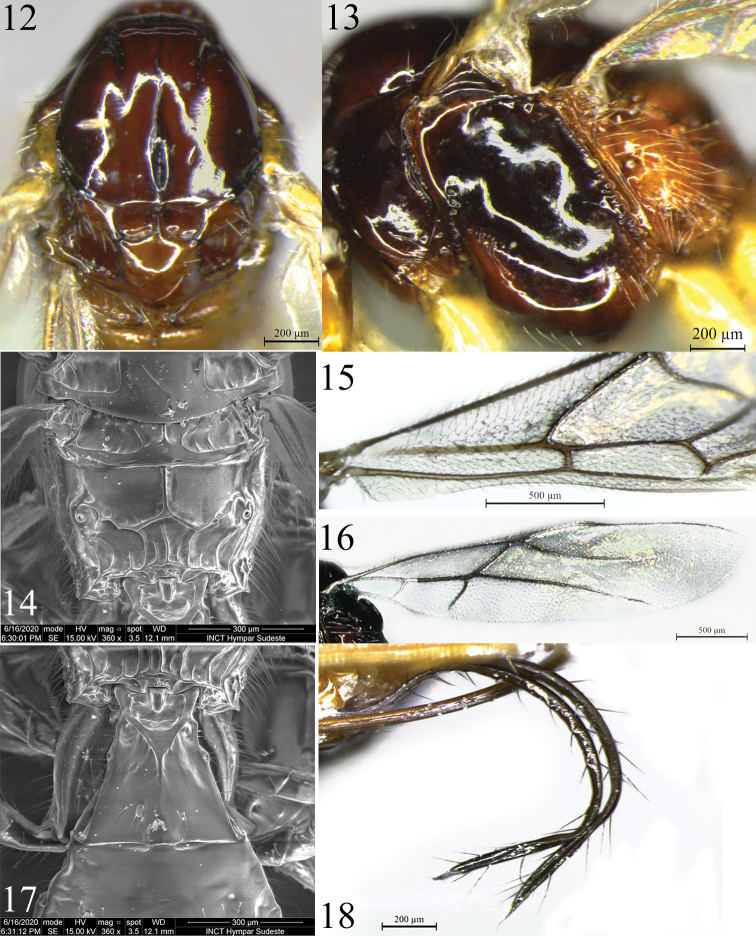
*Rhacalysia ampla* sp. nov. (females, paratypes) **12** mesoscutum and scutellar sulcus, dorsal view **13** mesosoma, lateral view **14** metanotum and propodeum, dorsal view **15** fore wing **16** hind wing **17** first metasomal tergite, dorsal view **18** ovipositor sheaths.

***Mesosoma*.** 1.3–1.4× as long as high, 1.9–1.95× as long as wide, 2.0–2.4× as high as head. Pronotum in dorsal view with distinct pronope, crenulate laterally; smooth in lateral view. Notauli deep, narrow, crenulate anteriorly, absent posteriorly (Fig. 12). Mesoscutum 1.05–1.1× as wide as long, scattered setae present along notauli. Scutellar sulcus 2.2–2.7× as wide as long, with well-developed mid ridge and smooth lateral areas. Mesoscutal pit deep, slightly elongate, occupying 1/6 to 1/5 extent of mesoscutum (Fig. 12). Scutellar disc smooth, setiferous; parascutellar area smooth to rugose posteriorly, with setae near scutellar sulcus. Metanotum setiferous anteriorly, in dorsal view smooth to rugulose medially and very weakly crenulate near posterior margin of depressed lateral fields; mid ridge well-developed anteriorly, absent posteriorly, lateral ridges absent (Fig. 14); metanotum in lateral view without high median flange. Mesopleuron with some setae in posterior area below, antero-basal margin crenulate towards anterior subalar area; posterior margin crenulate. Precoxal sulcus deep, crenulate weakly on anterior fourth of mesopleuron, mostly smooth (Fig. 13). Propodeum smooth, except for some rugae inside areola; anterior half with median carina, posterior half with pentagonal areola ca. as long as wide (Fig. 14). Metapleuron rugose posteriorly and setose.

***Fore wing*.** 0.9–1.1× as long as body. Pterostigma 3.6–3.75× as long as wide, 1.9–2.2× as wide as vein r length; r 0.2–0.3× as long as 2-SR, arising distad midpoint of pterostigma; submarginal cell 2.5–2.7× as long as high; 2-SR 2.5–2.7× as long as r-m, 1.1–1.3× as long as 3-SR; 3-SR 3.1–3.5× as long as r, 2.1–2.3× as long as r-m; SR1 3.5–4.0× as long as 3-SR; 2-CU1 1.3–1.4× as long as m-cu, this interstitial; cu-a postfurcal by distance ca. equal to its length; subdiscal cell closed, expanded distally, CU1a arising below middle of subdiscal cell (Fig. 15).

***Hind wing*.** With three hamuli, 1.3–1.4× as long as wide; vein 1-M 1.2× as long as M+CU, 1.3–1.6× as long as 1r-m; m-cu antefurcal, strongly nebulous for most of its length, tubular basally near its insertion, nearly reaching wing margin (Fig. 16).

***Legs*.** Hind femur 5.7–6.1× as long as wide. Hind tibia 11.4–12.1× as long as its maximum subapical width, 1.0–1.1× as long as hind tarsus. First segment of hind tarsus 1.5–1.7× as long as second segment.

***Metasoma*.** First metasomal tergite ca. as long as apical width; apex 2.0–2.1× as wide as base; smooth surface, dorsal carinae strongly convergent, uniting in basal third, continuing as distinct median carina but not reaching to apex; dorsope deep (Fig. 17). Ovipositor 1.0–1.25× as long as hind tibia, 1.1–1.4× as long as mesosoma; strongly curved upwards (Fig. 8). Ovipositor sheath setose (Fig. 18).

***Color*.** Mostly dark brown. Mandibles light brown to yellow, darker at base. Clypeus, scape, pedicel, scutellum, and metanotum brown to light brown. Flagellar segment 17–20 whitish (holotype). Mesonotum brown to reddish brown. Propodeum and metapleuron yellowish to dark orange. First metasomal tergite orange to yellowish orange, base of terga 2 sometimes orange, other tergites brown. Tegula, ovipositor, and most of legs yellow. Trochanter and tronchantellus pale yellow, telotarsus darkened; hind leg with distal tibia and tarsus brown. Wings hyaline; venation and pterostigma light brown to brown.

**Male.** Unknown.

#### Etymology.

The species name refers to the form of the paraclypeal fovea.

#### Distribution.

Brazil, State of Rio de Janeiro, Teresópolis, dense ombrophilous forest.

#### Comments.

*Rhacalysia ampla* is similar morphologically to *R. delicata*, with which it shares many characteristics. Members *Rhacalysia ampla* can be differing by the precoxal sulcus weakly sculptured only in the anterior fourth of mesopleuron (Fig. 13) (sculpture shallow but long in *R. delicata*), vein CU1a of fore wing arising below middle of subdiscal cell (Fig. 15) (at middle in *R. delicata*), and the following quantitative ratios: eye ca. 3.0× as wide as temples (2.2× in *R. delicata*); mesosoma 2.0–2.4× as high as head (1.7× in *R. delicata*); pterostigma 1.9–2.2× as wide as vein r length (3.0× in *R. delicata*); hind femur 5.7–6.1× as long as wide (5.0× in *R. delicata*); and ovipositor 1.0–1.2× as long as hind tibia (2.0× in *R. delicata*).

### Rhacalysia jatai sp. nov.

Taxon classificationAnimaliaHymenopteraBraconidae

10B71D3D-1D2B-512E-B3F9-D23562523E7B

http://zoobank.org/0D1B38F5-B74A-46DE-A61D-B56D95A28905

Figures 19–23 , 24–27

#### Type material.

***Holotype*** pinned, female, (DCBU 408525) Brazil, São Paulo, Luiz Antônio, Estação Ecológica do Jataí, 21°36'S, 47°47'W, 9.XI.2006, seasonal forest, Malaise trap, N. W. Periotto col. Original label: “Luiz Antonio/SP, EE. Jataí, 9/11/06, Col. N. Periotto”.

#### Diagnosis.

*Rhacalysia jatai* can be recognizable by the notauli and precoxal sulcus entirely smooth, fore wing with m-cu interstitial, CU1a arising at middle of subdiscal cell, hind wing with four hamuli; ovipositor 2.2× as long as mesosoma, body yellow (without brown parts).

**Figures 19–23.**
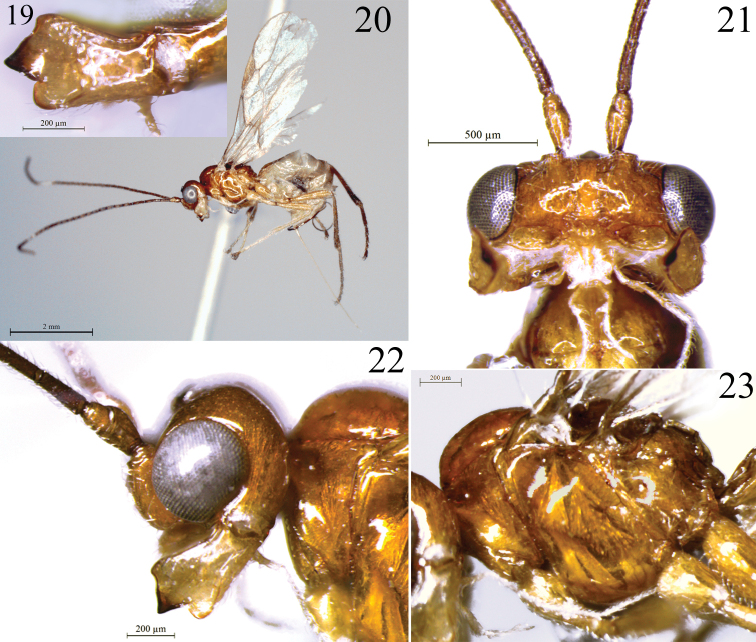
*Rhacalysia jatai* sp. nov. (female, holotype) **19** mandible, lateral view **20** habitus, lateral view **21** head and basal antennae, anterior view **22** head and pronotum, lateral view **23** mesosoma, lateral view.

#### Description.

**Female** (holotype) (Fig. 20). Length. Body 3.8 mm; fore wing 4.0 mm; hind wing 2.9 mm.

***Head*.** 1.7× as wide as long; 1.7× as wide as face, 1.6× as wide as mesosoma; 1.9 × as wide as apex of first metasomal tergite; slightly wider at eyes than temples in dorsal view. Eye glabrous, ca. as high as wide, 2.5× as wide as temple in lateral view (Fig. 22). Occiput, vertex, and temples smooth, with some sparse setae. Frons with weak pit mesally. Face 2.3× as wide as high, setose; low mid ridge dorsally and some weak transversal striae just above epistomal sulcus (Fig. 21). Epistomal sulcus deep, crenulate. Clypeus protruding, smooth, setose (setae as long as clypeus width), 1.6× as wide as high; lateral margin of clypeus does not contact with paraclypeal fovea. Malar space ca. 1/12 eye height. Paraclypeal fovea enlarged to form broad groove reaching to eye (Fig. 21). Mandible 3-dentate (Fig. 19), 1.6× as long as apical width, apex 1.5× as wide as base; setose, slightly rugulose medially; diagonal ridge well developed on apical half of mandible, ventral carina present on basal third of mandible; teeth 1 and 2 connected by flange, indistinct incision; tooth 3 rounded, slightly wider than tooth 1; tooth 2 wider and longer than others. Antenna with apical flagellar segments missing, 31 flagellar segments present. First flagellar segment 3.5× as long as wide; second flagellar segment 5.6× as long as wide, 1.6× length of first segment; third flagellar segment 4.9× as long as wide, 1.4× length of first segment. Maxillary palp ca. twice as long as head height.

**Figures 24–27.**
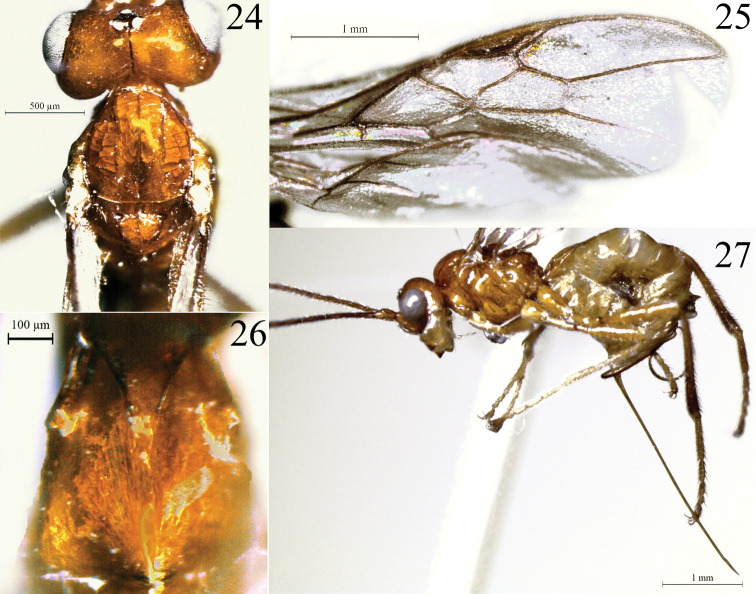
*Rhacalysia jatai* sp. nov. (female, holotype) **24** head, mesoscutum, and scutellar sulcus, dorsal view **25** fore wing **26** first metasomal tergite, dorsal view **27** habitus, lateral view showing ovipositor.

***Mesosoma*.** 1.3× as long as high, 2.0× as long as wide, 2.2× as high as head. Pronotum smooth (Fig. 22); in dorsal view, with some setae mesally, pronope absent. Notauli deep anteriorly but smooth, absent posteriorly (Fig. 24). Mesoscutum as wide as long, with scattered setae. Mesoscutal pit shallow, lightly elongate, occupying ca. 2/7 extent of mesoscutum. Scutellar sulcus 3.0× as wide as long, with well-developed mid ridge and some weak ridges at posterior margin of lateral areas. Scutellar disc and parascutellar area smooth, setiferous. Metanotum smooth, setiferous anteriorly; depressed lateral fields weakly crenulate in the posterior margin; mid ridge complete, lateral ridges absent; metanotum in lateral view with median flange slightly higher than scutellar disc. Mesopleuron smooth, with some sparse setae. Precoxal sulcus deep, entirely smooth (Fig. 23). Propodeum with median carina anteriorly; posterior half with pentagonal areola ca. as long as wide; rugulose inside areola, smooth remaining. Metapleuron smooth, setose.

***Fore wing*.** 1.05× as long as body. Pterostigma 4.0× as long as wide, 2.2× as wide as vein r length; r 0.2× as long as 2-SR, arising distad midpoint of pterostigma; submarginal cell 2.25× as long as high; 2-SR 2.4× as long as r-m, 1.3× as long as 3-SR; 3-SR 3.8× as long as r, 1.8× as long as r-m; SR1 3.8× as long as 3-SR; 2-CU1 1.2 × as long as m-cu; m-cu interstitial; cu-a postfurcal by distance slightly shorter than its length (Fig. 25); subdiscal cell closed, slightly expanded distally, CU1a arising near middle of subdiscal cell.

***Hind wing*.** With four hamuli, 4.4× as long as wide; vein 1-M 0.9× as long as M+CU, 1.6× as long as 1r-m; m-cu antefurcal, nebulous but heavily pigmented.

***Legs*.** Hind femur 5.2× as long as wide. Hind tibia 11.2× as long as its maximum subapical width, 1.1× as long as hind tarsus. First segment of hind tarsus 1.95× as long as second segment.

***Metasoma*.** First metasomal tergite 0.9× as long as apical width; apex 2.0× as wide as base; smooth surface, dorsal carinae converging anteriorly but not extending as median carina (Fig. 26); dorsope deep. Ovipositor 2.0× as long as hind tibia, 2.2× as long as mesosoma, straight (Fig. 27). Ovipositor sheath setose.

***Color*.** Yellow. Ocellar field and epicranial suture dark brown (Fig. 24); mandibles, fore coxae, and metasoma (except fist metasomal tergite) pale yellow. Flagellum light brown, except flagellar segments 19–21 whitish (holotype). Mesosoma dorsally and first metasomal tergite yellow-orange. Legs with telotarsus light brown; hind leg from tibia to apex and ovipositor sheath brown. Wings hyaline, venation and pterostigma light brown.

**Male.** Unknown.

#### Etymology.

The name of species refers to locality of collection of material for study.

#### Distribution.

Brazil, State of São Paulo, Luiz Antônio, seasonal forest.

#### Comments.

**Figures 28–34.**
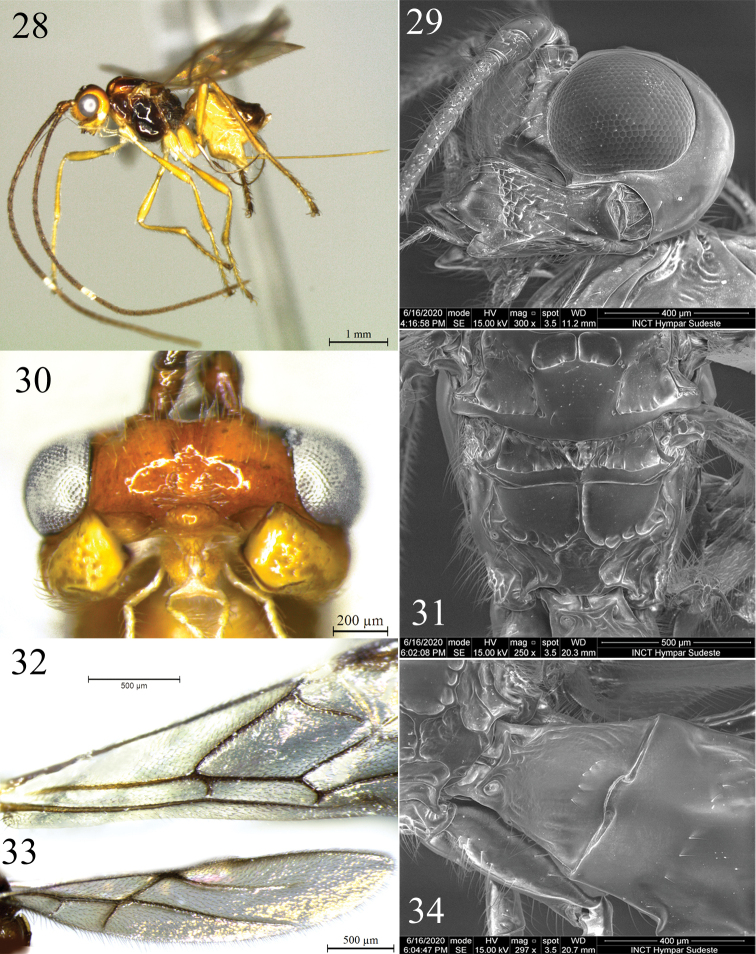
*Rhacalysia monteiroi* sp. nov. (females; **28** and **32** holotype, others paratypes) **28** habitus, lateral view **29** head, lateral view **30** head, anterior view **31** metanotum and propodeum, dorsal view **32** fore wing **33** hind wing **34** first metasomal tergite, posterior view.

**Figures 35–38.**
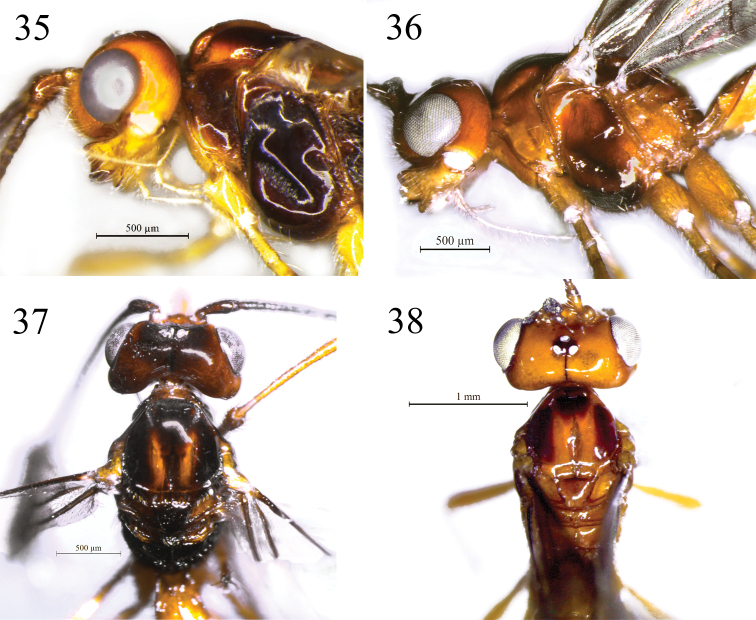
*Rhacalysia monteiroi* sp. nov. (females; **35** holotype, **36–38** paratypes) **35** head and mesosoma, lateral view, precoxal sulcus crenulate **36** head and mesosoma, lateral view, precoxal sulcus smooth **37** head and mesosoma, dorsal view **38** head and mesosoma, dorsal view.

*Rhacalysia jatai* is morphologically similar to *R. monteiroi* and both species shares the hind wing with four hamuli, as well as several other features. *Rhacalysia jatai* can be differentiated by the follow quantitative ratios: third flagellar segment 4.9× as long as wide (4.5–4.6× in *R. monteiroi*), 1.4× length of first segment (1.0–1.1 × in *R. monteiroi*); eye 2.5× as wide as temple (1.5–2.0× in *R. monteiroi*); vein 3-SR of fore wing 3.8× as long as r (3.0–3.1× in *R. monteiroi*); hind femur 5.2 × as long as wide (6.2–6.7× in *R. monteiroi*); hind tibia 11.1× as long as its maximum apical width (12.2–12.7× in *I. monteiroi*); ovipositor 2.0× as long as hind tibia (1.3–1.5 × in *R. monteiroi*), 2.2× as long as mesosoma (1.4–1.7× in *R. monteiroi*). In addition, in *Rhacalysia jatai* the body is entirely yellowish (Figs 24, 27), while in *R. monteiroi* the color pattern of mesosoma and metasoma is mixed between yellowish and distinctly brown parts (Figs 28, 35–38).

### Rhacalysia monteiroi sp. nov.

Taxon classificationAnimaliaHymenopteraBraconidae

56081D30-B52D-5D16-8D83-421EB991BB0F

http://zoobank.org/C9B2CD68-89AF-434D-87DF-03BEEC3B3303

Figures 28–34 , 35–38

#### Type material.

***Holotype*** pinned, female, (DCBU 404794) Brazil, Rio de Janeiro, Teresópolis, Parque Nacional da Serra dos Órgãos, 22°26'54"S, 43°00'49"W, alt. 1482 m, XII.2014, dense ombrophilous forest, Malaise trap, R. F. Monteiro col. ***Paratypes*** females (2), (DCBU 374756) 22°28'11"S, 43°00'05"W, alt. 868 m, VII.2015, Malaise trap, R. F. Monteiro col.; (DCBU 361820) 22°31'00"S, 43°00'23"W, alt. 252 m, XI.2015, Malaise trap, R. F. Monteiro col.

**Diagnosis.***Rhacalysia monteiroi* can be recognized by the notauli incomplete, fore wing with m-cu interstitial, CU1a arising at middle or slightly below middle of subdiscal cell, hind wing with four hamuli, hind femur 6.2–6.7× as long as wide, coloration of body mixed between yellowish and brown parts.

#### Description.

**Female** (Fig. 28). ***Length*.** Body 3.4–4.1 mm; fore wing 3.9–4.2 mm; hind wing 2.6–3.0 mm.

***Head*.** 1.7–1.9× as wide as long; 1.7–1.9× as wide as face, 1.4–1.6× as wide as mesosoma; 2.1× as wide as apex of first metasomal tergite; slightly wider at eyes than temples in dorsal view. Eye glabrous, 1.1–1.2× as high as wide, 1.5–2.0× as wide as temples in lateral view (Figs 35, 36). Occiput, vertex and temples smooth, with some sparse setae. Frons smooth or with weak pit mesally. Face 2.0–2.5× as wide as high, setose; low mid ridge dorsally, with rugulose pair of grooves near clypeus or some transversal striae just above epistomal sulcus (Fig. 30). Epistomal sulcus deep, crenulate. Clypeus protruding, smooth, setose (setae as long as clypeus width), 1.6–1.8× as wide as high; lateral margin of clypeus does not contact paraclypeal fovea. Malar space ca. 1/10 eye height. Paraclypeal fovea enlarged to form broad groove extending to eye. Mandible 3-dentate (Fig. 29), 1.6–1.7× as long as apical width, apex 1.4× as wide as base; setose, rugulose medially; diagonal ridge well developed on apical half of mandible, ventral carina not visible; teeth 1 and 2 connected by flange, indistinct incision; tooth 2 wider and longer than others. Antenna 1.9× as long as body, with 40 flagellar segments (holotype). First flagellar segment 3.3–3.8× as long as wide; second flagellar segment 5.2–5.8× as long as wide, 1.3× length of first segment; third flagellar segment 4.5–4.6× as long as wide, 1.0–1.1× length of first segment. Maxillary palp 2.25–2.55 × as long as head height.

***Mesosoma*.** 1.3–1.4× as long as high, 1.9× as long as wide, 2.4× as high as head. Pronotum with pronope relatively large, slightly crenulate laterally and with some setae in dorsal view; smooth to slightly crenulate in lateral view. Mesoscutum 1.1× as wide as long, scattered setae, smooth to weakly crenulate in postero-lateral margins. Notauli deep, smooth to weakly crenulate anteriorly, absent posteriorly (Figs 37, 38). Mesoscutal pit deep, elongate, occupying 1/4 to 1/3 extent of mesoscutum. Scutellar sulcus 2.6–3.0× as wide as long, with well-developed mid ridge and smooth lateral areas. Scutellar disc smooth, setiferous; parascutellar area smooth, with setae near scutellar sulcus. Metanotum setose anteriorly, in dorsal view rugose to rugulose medially, smooth to slightly crenulate near anterior and posterior margins of depressed lateral fields (Fig. 31); anterior mid ridge complete, some lateral carinae incomplete to absent; metanotum in lateral view with median flange slightly higher than scutellar disc. Mesopleuron with some setae postero-ventrally and subalar area; antero-basal margin weakly crenulate towards anterior subalar area; posterior margin crenulate. Precoxal sulcus deep, crenulate, separated from posterior margin (Fig. 35) or almost entirely smooth (Fig. 36). Propodeum with median carina anteriorly, posterior half with pentagonal areola ca. as long as wide (Fig. 31); smooth to slightly rugose near to carinae and inside areola. Metapleuron rugose posteriorly and setose.

***Fore wing*.** 1.0–1.2× as long as body. Pterostigma 3.6–4.1× as long as wide, 2.1–2.3× as wide as vein r length; r 0.2–0.25× as long as 2-SR, arising distad midpoint of pterostigma; submarginal cell 2.5–3.5× as long as high; 2-SR 2.4–2.7× as long as r-m, 1.3–1.6× as long as 3-SR; 3-SR 3.0–3.1× as long as r, 1.7–2.0× as long as r-m; SR1 3.8–4.0× as long as 3-SR; 2-CU1 1.0–1.35× as long as m-cu, this interstitial; cu-a postfurcal by distance ca. equal to its length; subdiscal cell closed, slightly expanded distally, CU1a arising at middle to slightly below middle of subdiscal cell (Fig. 32).

**Hind wing.** With four hamuli, 4.1–4.9× as long as wide; vein 1-M 1.0–1.2× as long as M+CU, 1.4–1.7× as long as 1r-m; m-cu antefurcal, heavily nebulous, tubular basally or not, almost reaching wing margin (Fig. 33).

***Legs*.** Hind femur 6.2–6.7× as long as wide. Hind tibia 12.2–12.7× as long as its maximum subapical width, 1.1–1.2× as long as hind tarsus. First segment of hind tarsus 1.7–1.8× as long as second segment.

***Metasoma*.** First metasomal tergite 1.0–1.1× as long as apical width; apex 1.8–1.9 × as wide as base, strigose to slightly strigose; dorsal carinae converging in basal third, extending posteriorly as median carina incomplete or median carina absent (Fig. 34); dorsope deep. Ovipositor 1.35–1.5× as long as hind tibia, 1.45–1.7× as long as mesosoma; straight. Ovipositor sheath setose.

***Color*.** Body parts vary between dark brown to yellow (Figs 35–38). Head mostly yellow, vertex yellow to brown, ocellar field and epicranial suture dark brown; mandible light yellow. Flagellar segments brown to dark brown, except 18–19 whitish (holotype). Propleuron yellow; mesonotum yellow-orange, with more or less developed lateral and antero-medial spots brown, other parts of mesosoma varying from yellow-orange to dark brown. Legs yellowish to orange; telotarsus brown; hind leg with tibia and tarsus darker. Metasoma yellow except for metasomal tergites 4 towards to apex and ovipositor sheaths brown. Wings hyaline to dusky, venation and pterostigma light brown to dark brown.

**Male.** Unknown.

#### Etymology.

The species is named in honor of Ricardo Ferreira Monteiro, the collector of the studied material.

#### Distribution.

Brazil, State of Rio de Janeiro, Teresópolis, dense ombrophilous forest.

#### Comments.

*Rhacalysia monteiroi* shares many morphological characteristics with *R. jatai*; both species can be differentiated by the coloration pattern of body, relative length of the ovipositor, and relative length/wide of the posterior femur (see above).

The three specimens of *R. monteiroi* studied here vary considerably in some characteristics. In short, the sculpturing of face (striate or rugulose above the clypeus), notauli (smooth or weakly crenulate), precoxal sulcus (smooth or crenulate), and first metasomal tergite (with or without median carina); the coloration of vertex, pronotum, metanotum, propodeum, and metapleuron yellowish or brown (Figs 35–38). Despite this, the observed variations were not significant to consider them as different species.

Members of the genera *Idiasta* and *Rhacalysia* can be morphologically differentiated as follows: paraclypeal fovea not extending to eye in *Idiasta* (Fig. 3); paraclypeal fovea enlarged to form broad groove extending to eye in *Rhacalysia* (Figs 10, 21).

### Key to the Neotropical species of the genus *Idiasta*

**Table:** 

1	Fore wing patterned with several dark spots or bands, M+CU1 of fore wing very weak, not, or only weakly, pigmented for much of its length; notauli complete to mesoscutal pit; metanotum with high flange. Body length 3.0–5.0 mm. Mexico, Holarctic	***I. maritima* (Haliday) (♀♂)**
–	Fore wing either hyaline or dusky but never patterned with spots or bands; M+CU1 of fore wing well-developed, usually strongly pigmented throughout; variable development of notauli; metanotum with or without high flange	**2**
2	Notauli complete and rugose; fore wing with CU1a arising well below middle of first subdiscal cell, cu-a interstitial or postfurcal; metanotum with high flange. Body length 2.5–4.0 mm. Mexico	***I. euryzona* Wharton (♀♂)**
–	Notauli incomplete, not reaching mesoscutal pit (as Fig. 12); fore wing with CU1a arising at middle or slightly above middle of subdiscal cell, cu-a postfucal; metanotum with or without high flange	**3**
3	Eye with scattered setae, maxillary palp 1.4× as long as head height; scutellar sulcus 1.4× as wide as long; metanotum without high flange; 3-SR of fore wing 3.4× as long as r, m-cu slightly antefurcal (2-SR+M present); ovipositor ca. as long as mesosoma. Colombia	***I. dixi* Dix (♀)**
–	Eye glabrous; maxillary palp 2.0–2.4× as long as head height; scutellar sulcus 2.2–2.5× as wide as long; metanotum with high flange; 3-SR of fore wing 2.6× as long as r, m-cu interstitial (2-SR+M absent); ovipositor 1.45× as long as mesosoma. Body length 2.4–2.7 mm. Brazil (Figs 1–7)	***I. rupina* sp. nov. (♀♂)**

### Key to the species of the genus *Rhacalysia*^1^

**Table:** 

1	Fore wing with vein CU1a interstitial. Body length 2.4 mm. Republic of the Congo.	***R. congoensis* Fischer (♀♂)**
–	Fore wing with variable CU1a insertion but not interstitial	**2**
2	Notauli complete and heavily sculptured; face with two wide and sculptured longitudinal sulcus lateral to mid ridge. Body length 4.3 mm. India	***R. rufobalteata* Cameron (♀♂)**
–	Notauli smooth or incomplete (Figs 12, 37, 38); face without long and sculptured sulcus (Figs 10, 21, 30)	**3**
3	Clypeus slightly wider than high; vein m-cu of fore wing antefurcal; scutellar sulcus with 7 longitudinal ridges; first metasomal tergite longitudinally striate, median carinae present; body entirely black. Body length 5.0 mm. India	***R. profundinigra* Fischer (♀♂)**
–	Clypeus 1.6–2.0× as wide as long; vein m-cu of fore wing interstitial (Fig. 25); scutellar sulcus with mid ridge, lateral carina absent (Fig. 12); first metasomal tergite smooth to strigose, with or without median carinae; body color variable.	**4**
4	Hind wing with three hamuli; metanotum without high flange; body color mostly brown.	**5**
–	Hind wing with four hamuli; metanotum with high flange; body yellowish or at least with distinctly yellow-orange parts.	**6**
5	Eye 2.0–2.2× as wide as temple; mesosoma 1.7× as high as head; hind femur ca. 5.0× as long as wide; fore wing with pterostigma 3.0× as wide as vein r length, cu-a slightly postfurcal, CU1a arising at middle of subdiscal cell; ovipositor ca. twice as long as hind tibia. Body length 3.5–5.4 mm. Argentina, Brazil, Colombia, Mexico, Panama, Peru, and Venezuela	***R. delicata* (Papp) (♀♂)**
–	Eye ca. 3.0× as wide as temple; mesosoma 2.0–2.4× as high as head; hind femur 5.7–6.1× as long as wide; fore wing with pterostigma 1.9–2.4× as wide as vein r length, cu-a postfurcal by distance ca. equal to its length, CU1a arising below of subdiscal cell; ovipositor 1.0–1.2× as long as hind tibia. Body length 3.1–4.2 mm. Brazil (Figs 8–18)	***R. ampla* sp. nov. (♀)**
6	Vein 3-SR of fore wing 3.8× as long as r; hind femur 5.2× as long as wide; hind tibia 11.2× as long as its maximum subapical width; ovipositor 2.0× as long as hind tibia, 2.2× as long as mesosoma; body color yellowish. Body length 3.8 mm. Brazil (Figs 19–27).	***R. jatai* sp. nov. (♀)**
–	Vein 3-SR of fore wing 3.0–3.1× as long as r; hind femur 6.2–6.7× as long as wide; hind tibia 12.2–12.7× as long as its maximum subapical width; ovipositor 1.4–1.5× as long as hind tibia, 1.5–1.7× as long as mesosoma; mesosoma with distinctly brown parts. Body length 3.4–4.1 mm. Brazil (Figs 28–38)	***R. monteiroi* sp. nov. (♀)**

## Discussion

The wing venation pattern of *Idiasta* is widely maintained in *Rhacalysia*. The morphological support for the retention of the generic status of *Rhacalysia* has been the enlarged paraclypeal fovea (Fischer 1967, 1994; Wharton 1980, 2002). However, although it is decidedly an apomorphic character within Alysiinae, it is not clear that all species with the enlarged paraclypeal fovea form a monophyletic group (Wharton 2002).

The insertion antefurcal of vein m-cu of fore wing (and therefore 2-SR+M present) was considered diagnostic characteristic of *Idiasta* by Wharton (2002), supposedly differing from the trend observed in *Rhacalysia* species (this vein less antefurcal). Indeed, m-cu of fore wing is interstitial in most known *Rhacalysia* species but is antefurcal in *R. congoensis* and *R. profundinigra*. Moreover, this vein is interstitial in *I. rupina* (Fig. 6) and postfurcal in Apiasta Wharton, 2002, a subgenus of Idiasta known from Australian Region and considerably similar to *Rhacalysia* in morphology (Wharton 2002). It has also been argued that the density of setae on the ovipositor sheath is typically higher in *Idiasta* than *Rhacalysia* (Wharton 1980, 2002). Here, we observed that the setae ovipositor sheath in *R. ampla*, *R. jatai*, and *R. monteiroi* are separated by a distance shorter than its length (Fig. 18), although sparser than *I. rupina*. Thus, this feature must be used carefully.

In addition to the enlarged paraclypeal fovea, all known species of *Rhacalysia* shares the follow characteristics: fore wing with pterostigma distinct and wide, vein r arising from its distal middle, vein r shorter than pterostigma width, cu1 postfurcal (Figs 15, 25, 32); mid ridge present on face (at least weakly developed); and first metasomal tergite widened towards to apex (apical width ca. twice the basal) (Fig. 17). Furthermore, the vein CU1a of fore wing not interstitial, m-cu of hind wing well-developed, and indistinct incision between mandibular teeth 1 and 2 (Figs 11, 19, 29), are characteristics shared by all species except *R. congoensis*, with relatively distinct morphology, in which the CU1a of fore wing is interstitial, m-cu of hind wing absent, and there is well defined incision between teeth 1 and 2.

Studies covering more *Rhacalysia* specimens in the future, should provide a clearer and more conclusive morphological delineation in relation to the genus *Idiasta*, especially if together the analysis of molecular data. Nevertheless, as well as Fischer (1994) and Wharton (2002), we maintain the genus position of *Rhacalysia*, and consider *R. delicata* part of it, based on the new species described here.

Considering our records, the distribution of *Idiasta* is slightly altered: the genus is no longer recorded from Argentina, Peru, and Venezuela. In turn, considering *delicata* species as *Rhacalysia*, the genus *Rhacalysia* is now known from many countries of the Neotropical Region.

## Supplementary Material

XML Treatment for Idiasta

XML Treatment for Idiasta rupina

XML Treatment for Rhacalysia

XML Treatment for Rhacalysia ampla

XML Treatment for Rhacalysia jatai

XML Treatment for Rhacalysia monteiroi

## Footnotes

Modified from Fischer 1999.
